# The College of Nursing (Limited by Guarantee)

**Published:** 1920-10-30

**Authors:** 


### The College of Nursing
### (Limited by Guarantee).

Notice is hereby given that an Extraordinary General Meeting of the College of Nursing, Limited, will be held at the Royal Society of Medicine, 1 Wimpole Street, W. 1, on Thursday, November 4, 1920, at 4 o'clock in the afternoon, when the subjoined Resolutions will be proposed.

Resolutions.

1. That Article 6 of the Articles of Association be cancelled and the following Article substituted in its place:—

6. A person appointed to be a member of the Council (including therein the President appointed by Clause 34 hereof) shall by virtue of such appointment become forthwith a member of the College. Except the subscribers hereto, no person shall be capable of being a member unless he be a member of the Council, or of a Local Board, or be upon the Register. A person qualified under Section 3 (1) of the Memorandum of Association may be placed upon the Register and become a member of the College by paying an entrance fee of one pound and one shilling, and by and on signing such form of application for registration and membership, and agreement to be bound by the Memorandum and Articles of Association and Regulations of the College, as the Council may from time to time require. Every member shall pay such annual subscription not being more than twenty shillings as the Council shall from time to time appoint. He may at any time pay to the Council such a sum as with the subscriptions already paid by him (not exceeding ten) equals twenty annual subscriptions at the date of such payment, and thereupon his annual subscriptions shall cease and he shall become a Life Member of the College. If his name be removed from the Register he shall cease to be a member, but not otherwise.

No member registered after November 20, 1920, shall enjoy any of the privileges of membership until he has paid his annual subscription and any arrears thereof.

No member registered after November 20, 1920, shall vote at any annual meeting unless he has paid his annual subscription and any arrears thereof at least ten days before such annual meeting.

The Council may order, if it shall think fit, the removal from the Register of the name of any member registered after November 20, 1920, who is in arrear with his subscriptions for five years or upwards.

2. That the following words be added to Article 60, Clause 9:—and that at least twenty-eight days' previous notice of the meeting and its purpose shall be sent to the member with regard to whom an inquiry is to be held, and he shall have an opportunity of stating his case and defending himself before the Council.

Should the above Resolutions be passed by the requisite majority they will be submitted for confirmation as Special Resolutions to a further Extraordinary General Meeting, which will be held on Saturday, November 20, 1920, at 3 p.m., at the Royal Society of Medicine, 1 Wimpole Street, W. 1.

By Order of the Council.

M. S. Rundle, Secretary.

### BIRMINGHAM AND THREE COUNTIES CENTRE.

As we go to press we learn that on Thursday, October 28, at 5.30 p.m., in the Lecture Theatre at the General Hospital, Birmingham (by kind permission of the Governors), Dr. D. K. Wilkinson will lecture on "Some Diseases of the Heart."

All nurses in the three Counties (Warwickshire, Worcestershire, Staffordshire) are reminded that the second number of *The Herald*, published in connection with the Scenic Fair, may be obtained from Mrs. Richards, Room 48, No. 3 New Street, Birmingham.

### THE NEW CENTRE AT TORQUAY.

A meeting in connection with the College of Nursing was held, by the kindness of the Matron, Miss V. Billing, R.R.C., at the Torbay Hospital on October 11. Miss Sheriff-MacGregor, Organising Secretary, addressed the meeting, and defined the aims of the College and its relationship in connection with State Registration. After several questions were put and answered, the members unanimously agreed to form a Torquay and District Centre. Mrs. Clutton was elected, and kindly consented to act as President, Miss Flemyng as Hon. Secretary, and Miss Turner as Hon. Treasurer. Six members were duly elected to serve on the Executive Committee.

It was decided to hold a monthly meeting during the coming months, the first to be of a social character, as a means of getting to know each other. It is hoped that some lectures on various topics will be given at the succeeding meetings.

